# Preparing the Next Generation of Integrative Organismal Biologists

**DOI:** 10.1093/icb/icae098

**Published:** 2024-07-05

**Authors:** Dianna K Padilla, Daniel Grünbaum

**Affiliations:** Department of Ecology and Evolution, Stony Brook University, Stony Brook, NY 11794-5245, USA; School of Oceanography, University of Washington, Seattle, WA 98195-7940, USA

## Abstract

Pursuing cutting edge questions in organismal biology in the future will require novel approaches for training the next generation of organismal biologists, including knowledge and use of systems-type modeling combined with integrative organismal biology. We link agendas recommending changes in science education and practice across three levels: Broadening the concept of organismal biology to promote modeling organisms as systems interacting with higher and lower organizational levels; enhancing undergraduate science education to improve applications of quantitative reasoning and modeling in the scientific process; and K-12 curricula based on Next Generation Science Standards emphasizing development and use of models in the context of explanatory science, solution design, and evaluating and communicating information. Out of each of these initiatives emerges an emphasis on routine use of models as tools for hypothesis testing and prediction. The question remains, however, what is the best approach for training the next generation of organismal biology students to facilitate their understanding and use of models? We address this question by proposing new ways of teaching and learning, including the development of interactive web-based modeling modules that lower barriers for scientists approaching this new way of imagining and conducting integrative organismal biology.

## Introduction

Organismal biology has undergone a sea change in the past 15 years, resulting in a fortunate synergy between K-12 education (Core Ideas, Scientific and Engineering practices, and Crosscutting Concepts defined by the Next Generation Science Standards, NGSS); undergraduate education (Core Competencies for biologists identified by the American Association for the Advancement of Science, AAAS's Vision and Change document) and research (grand challenges in organismal biology as articulated by the Society for Integrative and Comparative Biology, SICB and others). We need to take advantage of this synergy to train the next generation of organismal biologists.

### Grand challenges in organismal biology

During President Barack Obama's first term, the White House Office of Science and Technology Policy called upon companies, research universities, foundations, and philanthropists to join him in identifying and pursuing the grand challenges of the 21st century. Grand challenges are ambitious but achievable goals that will harness science, technology, and innovation to solve important national or global problems, catalyzing advances in science, technology, and other national priorities while capturing the public's imagination to gain support. Organized primarily through the SICB, discussions by organismal biologists culminated in a paper (Schwenk et al. 2009, shared first authorship) articulating five grand challenges in organismal biology: (1) understanding the organism's role in organism–environment linkages; (2) utilizing the functional diversity of organisms; (3) integrating living and physical systems analysis; (4) understanding how genomes produce organisms; and (5) understanding how organisms walk the tightrope between stability and change.

This paper spurred a wide range of scientists and educators to create new research foci addressing scientific and societal needs in the 21st century. In a 2010 SICB workshop Implementation of Grand Challenges in Organismal Biology, two major priorities were identified: (1) research areas that involve interdisciplinary cooperatives to move beyond standard model organisms, methodologies, and data analyses; and (2) education at all levels (high school, undergraduate, graduate, post-doctoral, and tenure-track faculty), and communicating the importance of organismal biology to legislators, granting agencies and the public. This began a national discussion among organismal biologists of ideas and recommendations in white papers, workshops, and published articles (e.g., Tsukimura et al. 2010; Stillman et al. 2011). Further workshops specifically focused on a research agenda for addressing the grand challenge question, understanding how organisms walk the tightrope between stability and change, resulting in a white paper, an article published in BioScience (Padilla et al. 2014), and the Organismal Systems Modeling Research Coordination Network (OSyM).

Padilla et al. (2014) articulated the importance of understanding living organisms as multiscale modular systems interacting as dynamic networks in time and space that require modeling approaches from mathematics and engineering to provide insights into stability and change, including the causes and effects of phenotypic plasticity and sensitivity of organisms to changing environments. In particular, Padilla et al. (2014) focused on the potential of predictive systems-type models to address central questions in areas such as climate change impacts, human health, and the fundamental mechanisms regulating evolutionary processes. In this context, “systems-type models” are models whose conceptualization and implementation reflect the structural and functional relationships both within and across organizational levels (e.g., organs within organisms; organisms within habitats), used to quantitatively understand and predict aggregate dynamical properties. Systems approaches are traditionally more common in molecular biology and ecosystems studies than in organismal biology (Brownell et al. 2014). Organisms lie in the middle of a continuum from smaller size and faster processes (molecules, genes, cells, chemical interactions) to the larger size and slower scale of ecosystems (size and structure of populations, impacts of biodiversity, movement, and balance of nutrients in a system; Brownell et al. 2014). Organismal traits and processes (e.g., physiology, phenotype, responses to the environment) are less frequently approached with systems models or thinking. Padilla et al. (2014) noted that it will require training new organismal biologists in new ways of thinking and asking questions to implement effective uses of systems-type models, and, more broadly, to incorporate modeling as a core method in organismal biology (see fig. 1 in Padilla et al. 2014). We are still in the early stages of determining how best to approach this training.

**Fig. 1:**
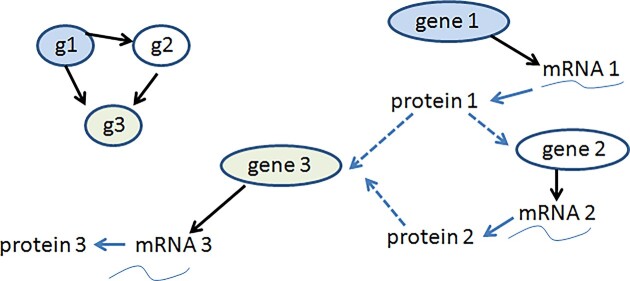
Example of a hypothetical gene network. The interactions of individual genes and gene products (mRNAs and proteins) are needed to understand the functioning of a cell.

### Vision and Change

Concurrent with discussions of grand challenges in organismal biology, educators from across the country were developing new ideas about more effective ways to teach science at the undergraduate level. In 2009, more than 500 biology educators and other stakeholders attended a national conference titled “Transforming Undergraduate Biology Education: Mobilizing the Community for Change.” As a result of this conference and continued dialog, the AAAS published Vision and Change in Undergraduate Biology Education, A call to action, identifying core concepts essential to biological literacy: (1) Evolution and the diversity of life-forms that have evolved over time through mutations, selection, and genetic change; (2) structure and function, including the basic units of biological structures that define the functions of all living things; (3) information flow, exchange and storage, including the influence of genetics on the control of the growth and behavior of organisms; (4) pathways and transformations of energy and matter, and the ways by which chemical transformation pathways and the laws of thermodynamics govern the growth and change of biological systems; and (5) systems, which includes the ways by which living things are interconnected and interact with one another (American Association for the Advancement of Science Vision and change in undergraduate biology education: a view for the 21st century, www.visionandchange.org [accessed 12 /12/2023], Brewer and Smith 2011) ). Vision and change also identified core competencies that are required for biological literacy and to practice science (1) the ability to apply the process of science; (2) the ability to use quantitative reasoning; (3) the ability to use modeling and simulation; (4) the ability to tap into the interdisciplinary nature of science; (5) the ability to communicate and collaborate with other disciplines; and (6) the ability to understand relationships between science and society. Thus, Vision and Change reflected a broad consensus among educators that undergraduate biology students need more experience with modeling, simulation, systems-level approaches, and use of large databases.

### NGSSs and a framework for K-12 science education

At about the same time, the National Academies and National Research Council recommended comprehensive changes in K-12 science education (in A framework for K-12 science education. Practices, crosscutting concepts, and core ideas; National Research Council 2012), which are generally known as the NGSS. NGSS emerged from an increased demand for standards for teaching K-12 science in the US to reflect recent advances education, science, and engineering. These new ways of teaching rely on what are defined as three dimensions of learning, each of which prioritizes the importance of quantitative reasoning: Core ideas are field-specific, many of which involve quantitative critical thinking. Scientific and engineering practices cross all fields and include development and use of models; analyzing and interpreting data; using mathematics and computational thinking; and obtaining, evaluating, and communicating information. Crosscutting concepts include identification of patterns; determining cause and effect through mechanisms and explanations; scale, proportion, and quantity; systems and systems models; energy and matter flows, cycles and conservation; structure and function; and stability and change.

### Training the new generation of organismal biology students

As noted by Clemmons et al. (2020), Vision and Change and NGSS align in addressing many of the same issues with similar priorities, especially with regard to the importance of quantitative reasoning, models, and systems approaches. If recommendations from NGSS and Vision and Change are followed, young scientists, especially upper level undergraduates and graduate students, will be better equipped to think about and use systems models to address questions in integrative organismal biology. Increased computer power now available to students, especially open source and cloud-based resources that foster interactions by students at all levels with active learning curricula designed by subject matter experts, will also greatly facilitate their ability to use quantitative thinking implemented in systems models to explain natural phenomena and understand their context in fundamental biological principles. Thus, we propose some new strategies for teaching the next generation of researchers in organismal biology that will take direct advantage of the new ways of teaching and learning from K-12 through undergraduate studies. In particular, we highlight the development of training modules that will enable new scientists to approach new ways of imagining and conducting integrative organismal biology studies, particularly studies that use systems models.

### Organism-centric models are the nexus between molecules and ecosystems

We view systems-type models centered around organisms as the nexus integrating higher and lower levels of organization. An illustrative concrete example comes from plankton dynamics in rapidly changing polar oceans. The pteropod *Limacina helicina antarctica* and its congeners are numerically and trophically important components of many marine food webs. Pteropods capture diverse phytoplankton and other small suspended organic particles using mucus net suspension feeding and are themselves key prey for diverse zooplankton, fish, seabirds, and mammals. Pteropods have thin aragonite shells and are regarded as indicator species for ocean acidification and other stresses. They contribute substantially to oceanic carbon sequestration by producing rapidly sinking fecal pellets and shells (Knecht et al. 2023).

The key roles of pteropods in marine food webs, and the imminent possibility that ocean change will dramatically alter those roles, have motivated studies of pteropods across scales from gene expression to biogeochemical dynamics. To cite a few recent examples: Gardner et al. (2023) investigated *L. h. antarctica*’s life history and population structure in the Southern Ocean. Johnson et al. (2019) measured near-ice seasonal transitions in the expression of genes indicative of maturation and reproduction, associated with environmental signals such as increased temperature, light, and food availability. Miller et al. (2023) investigated morphological features involved in protecting pteropod shells from ocean acidification-induced dissolution. Conroy et al. (2020) quantified diel vertical migration by *L. h. antarctica* in response to vertical distributions of phytoplankton. Spatial and temporal variations of water temperature, photosynthetically active radiation, phytoplankton productivity, and many other pertinent environmental parameters are key results of biogeochemical models of polar regions, which integrate ocean physics with fluxes of limiting nutrients such as nitrogen, carbon, and iron through plankton functional types such as viruses, bacteria, and subclasses of phytoplankton and zooplankton (Aumont et al. 2015; Ismail and Al-Shehhi 2023).

Organism-centered systems models have potential to integrate across all these diverse scales, for example by quantifying the effects of vertical movements on exposure to variable ambient conditions and environmental stresses to predict changes in gene expression and physiology that determine effects on reproduction and population dynamics. Furthermore, the motivations for many of these studies implicitly anticipates their application in integrated systems models that can exploit their quantitative results to obtain broader interpretations and predictions: A justification for investigating *A* because it has important impacts on *B* or is an important consequence of *C* implies that, now or in the foreseeable future, a method exists for making these connections explicit and quantitative.

Empowering future organismal biologists with the motivation and skills to undertake integrative modeling of this type is challenging but, in our view, feasible and necessary. Two perspectives underlie our optimism. First, as we discuss in more detail below, platforms for collaborative interactive modeling have progressed dramatically in recent years, greatly relaxing requirements for computing power and coding skills. Secondly, many of the most useful systems models, from gene networks to climate change, are bewildering and complex at first glance, but on closer examination are aggregations of simple, easy to understand parts (Fig. 1).

**Fig. 2:**
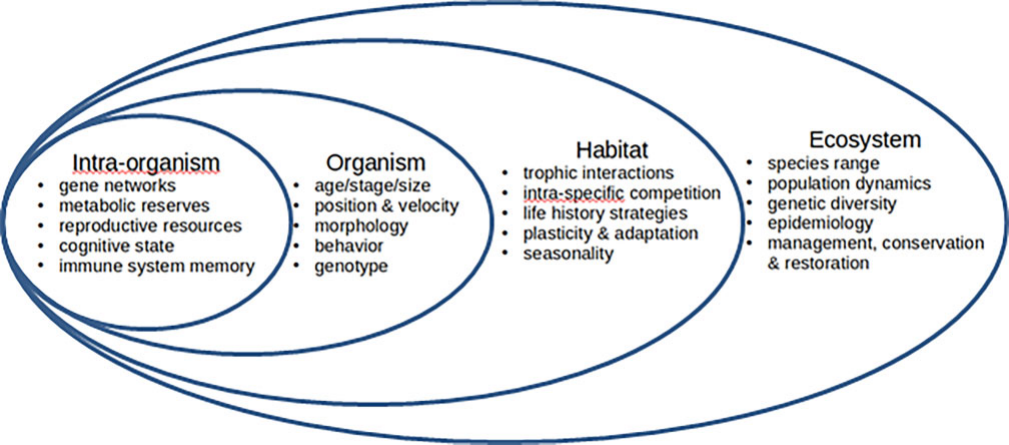
Within organisms are a variety of nested interaction networks that include genetic networks, physiological performance, development, and other processes. Organisms develop phenotypes, have behaviors and form populations. They are found within habitats that then affect those internal processes, and result in feedbacks with other organisms within a habitat. Local environments and habitats are similarly nested within ecosystems that influence responses of local systems as well as feedbacks on the larger scale processes.

For example, fluxes of limiting nutrients between plankton functional types in biogeochemical models used in climate prediction are typically governed by simple Monod regulatory mechanisms, where nutrient uptake or the intensity of a trophic interaction is specified by a maximum rate and an initial slope parameter (Aumont et al. 2015). The Monod regulatory functions (essentially equivalent to Michaelis–Menton functions) are intuitive, and to a trained student would provide a straightforward path to implement laboratory and field observations within the context of community-level models. Pursuing the example above, a biologist could embed a *L. h. antarctica* organism-specific model within a biogeochemical circulation model, and then use the temporal progression of ambient conditions during vertical migration to understand the dynamics of gene networks impacting reproduction (Fig. 2).

### Towards improved teaching of quantitative skills for organismal biologists

We note three strategies that we believe would help empower the next generation of organismal biologists to use modeling more broadly and with greater impact.

Organismal biology students should be taught why quantitative critical thinking skills generally, and mathematical modeling in particular, are useful and empowering in their own research. Many biology students do not regard modeling as relevant to their studies because they do not recognize ways that modeling is already embedded in their daily work. For example, statistical hypothesis tests are based on models of outcome probabilities. Many measurement techniques are based on sensors that use electrical characteristics as proxies, converted to environmental parameter values through models. More focused instruction on how to understand and use models, including models already part of routine scientific activities or that students read about in their everyday lives, would increase student effectiveness both as biological specialists and as communicators to other scientists and citizens. Students who engage in translating from a heuristic or conceptual model to a mathematical one are forced to consider specific assumptions at a level of detail that almost invariably, in our experience, reveals gaps in their understanding of driving mechanisms and the parameters that underlie those mechanisms. Simply writing mathematical models, even if they are never solved, clarifies students’ thinking about their organism, even in systems they know well. Models are also good tools for inferring characteristics of biological systems that we cannot measure. For example, mortality rates of most organisms are difficult or impossible to measure directly in the field; they typically must be inferred using models from population-level changes. Models offer students a way to specify biological quantities and processes in a language-independent way, communicating precise meanings among specialists in different scientific disciplines who often have different interpretations of the same terminology. Finally, empirical work on organisms is often confined to small windows in time at particular locations, and requires resources for travel, equipment, sample storage, etc. Discovery through modeling is essentially free, and is available anytime, anywhere.

Students should be introduced to recent open source online platforms that lower barriers to entry for high-level biological modeling. The scientific computing community has helped stimulate development of free, open-source tools that transform the ways by which numerical models can be developed, shared, and used in organismal biology teaching and research. An example is the coding language Python (https://www.python.org/) that is designed to be intuitive and easy to learn, but is nonetheless suitable for many advanced computer modeling and data analysis tasks. Python is an interpreted language (in contrast to compiled languages such as C or FORTRAN), designed to be used in interactive sessions that facilitate students’ understanding of cause and effect in modeled organismal systems. Jupyter notebooks (https://jupyter.org/; Kluyver et al. 2016) provide a free platform for embedding executable Python code (along with the statistical package R and many other computational resources) within browser pages that provide students with background, instructions, use cases, and links to additional resources. Jupyter notebooks can be stored and disseminated using a free GitHub account (https://github.com/). Models shared as Jupyter notebooks on GitHub can be executed in the cloud, again for free, on Binder (https://mybinder.org/). The Executable Book Project provides free utilities to compile assemblages of Jupyter notebooks and other content into electronic books containing functioning numerical models (see https://executablebooks.org/en/latest/gallery/for examples). Cumulatively, these and related developments in software and online resources provide a venue, free to anyone with an internet connection, to access knowledge, in the form of executable models, of mechanisms, parameters, concepts, and key questions shared by specialists in diverse areas of biological research.

Students should be taught how scaling analysis, nondimensional numbers, and dynamic similarity enable them to assess and predict dominant mechanisms affecting organisms. Scaling analysis is the study of how organismal characteristics, and the relative importance of mechanisms controlling their dynamics, change in proportion to basic parameters such as size (for example, body mass as the cube of length). Nondimensional numbers are ratios of parameter combinations with similar units. For example, in epidemiology, the basic reproduction number


\begin{eqnarray*}
{R_0} = \frac{{\text{number} \,\,\text{of} \,\,\text{new} \,\,\text{infections}}}{{\text{infectious} \,\,\text{cases}}}
\end{eqnarray*}

is nondimensional because the denominator and the numerator have the same units. Estimates of ${R_0}{\mathrm{ \,\,}}$ involve factors such as the number of contacts between contagious and susceptible hosts, and the probability of infection during each contact (Mahase 2020). Because ${R_0}{\mathrm{ \,\,}}$ is the key parameter determining onset of an epidemic in many models, different disease scenarios with the same reproduction number are dynamically similar—that is, they follow the same time course and intensity, when appropriately scaled. Informative nondimensional numbers are found in all areas of organismal biology, and are a basis for interpreting and predicting complex dynamics in a tractable and intuitive way. For example, the Reynolds number (*R*), an indicator of the relative magnitudes of inertial and viscous effects, is a key parameter in biomechanics. The drag on a swimming or flying organism is a complex function of many variables; however, it can often be neatly encapsulated as a function of *R* (Vogel 2013). Likewise, the effective availability of patchy resources to foraging animals can be estimated using the Frost number,


\begin{eqnarray*}
Fr = \frac{{\textit{search} \,\,\textit{time}}}{{\textit{patch} \,\,\textit{duration}}}
\end{eqnarray*}

(Urmy et al. 2022). Because it involves organismal traits such as movement speed and turning rate along with resource patch characteristics, this nondimensional number suggests organism-level requirements for successful foraging in different environments. In each of these examples, nondimensional numbers provide two key benefits: They condense multiple parameters into a much smaller set of mechanistically meaningful indicators, and they provide guidance for relating dynamically similar organismal systems and concisely summarizing their properties.

## A proposed agenda for organismal biology teaching

How could these three strategies for improved quantitative skills teaching contribute to training a more quantitatively empowered generation of students and to a more impactful and integrative future for organismal biology? We consider this question in light of traditional teaching approaches to quantitative critical thinking. We regard these approaches as admirable in concept but we believe that they have, in application, fallen short of their goals. These traditional approaches are typically rooted in training in relevant areas of mathematics (e.g., calculus, differential equations, linear algebra) and computer techniques (e.g., coding, numerical analysis). Coursework in these topics, together with disciplinary knowledge, provides students with the background to conceive and implement mathematical models of organismal systems that are rigorous and informative. The failure of this approach lies in the facts that too few organismal biology students pursue training in quantitative skills through this whole progression, and that the most useful content comes only at the end of the curriculum. For example, mathematical training almost invariably proceeds from algebra and functions, to calculus, linear algebra, ordinary differential equations (relating rates and states as functions of a single independent variable, such as time) and finally partial differential equations (relating rates and states as functions of multiple independent variables, such as time and space). This sequence reflects the order of technical difficulty—solving partial differential equations is the most difficult, and requires mathematical tools from ordinary differential equations, linear algebra, and calculus.

From a biologist's perspective, however, this order is backwards. Natural systems occur, and humans perceive them, as functions of multiple independent variables. Chemical concentration within tissue, morphological changes through development or during movement, immune system responses, population distribution, and countless other topics in organismal biology are inherently functions of time, space, size, stage, and other factors—in other words, they are most straightforwardly described by partial differential equations. It is through further layers of abstraction, for example, estimating infections by an implicit model for contact rates, or discretizing time into day- or year-long steps, that an intuitive but mathematically challenging partial differential equation scenario is simplified into an ordinary differential equation or matrix model that is more tractable but less directly comparable to the real world.

The vast majority of organismal biology students do not complete this quantitative training, because it has high opportunity cost and little reward until the conclusion of a long and difficult sequence. The traditional quantitative training, therefore, defers its benefits to an endpoint that most students never reach and asks the least prepared students to render their biological intuition into the most simplified abstraction.

We believe that these elements—improved understanding of the ways models can be useful; low-barrier, informative online platforms for high-level biological models; and mechanistic thinking skills using nondimensional numbers—offer a strategy to flip quantitative training for organismal biologists so that it is more intuitive and immediately useful. In particular, executable books and models embedded in online Jupyter notebooks provide a way for organismal biology students to experiment with models to gain quantitative skills and intuition, without regard to the complexity of the underlying mathematical techniques. We see this as analogous to “Driver's Ed” for modeling—just as most people learn to use cars safely and effectively with minimal knowledge of the internal mechanisms of car engines, organismal biology students could learn to apply models in rigorous and informative ways without a detailed understanding of code or computational techniques.

**Fig. 3:**
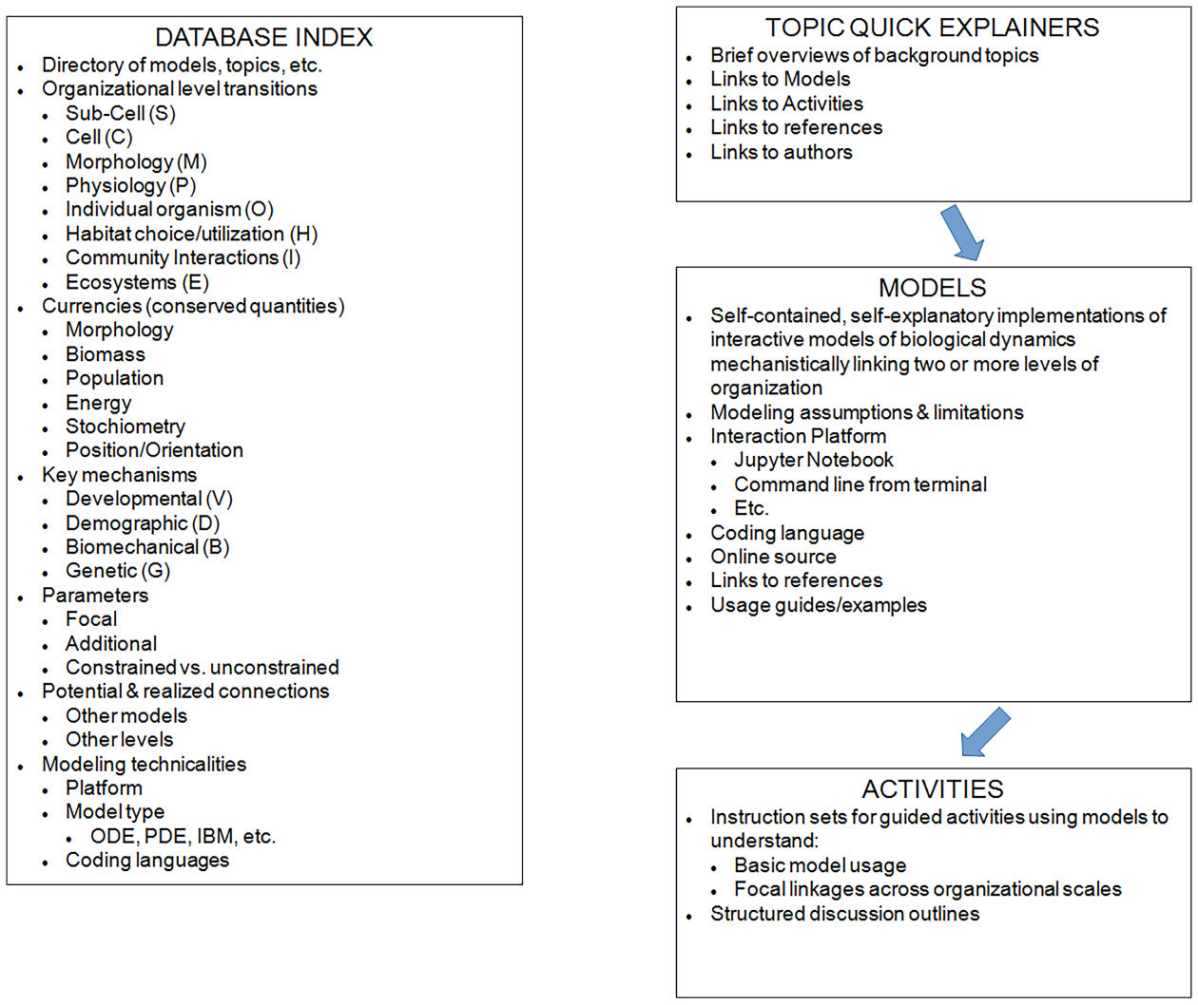
Conceptual structure for a library of online models, designed to function as a community-level distributed resource to facilitate experiential learning and quantitative research by organismal biologists. The key elements are a searchable database index that organizes library entries to facilitate finding models, activities, and background relevant to a given organizational level, organism, conserved quantity, mechanism, or computational platform of resources for students. Other elements of this online resource would include executable models, activities, and “quick explainers” of topics, which are brief summaries of jargon terms, concepts, or methods referred to in the executable models or activities.

We propose to implement this strategy with a library of community-level online models implemented in Python, presented with context in Jupyter notebooks and described in executable electronic books or equivalent documents (Fig. 3). Specifically, we envision a curriculum where students identify a question of interest, and work with online models to develop insights into the roles of key mechanisms that are important in addressing that question. This curriculum would emphasize enabling organismal biology students to develop the ability to

(i) use and conceptually understand (but not program) different modeling approaches;

(ii) use models to construct and test quantitative hypotheses about important mechanisms;

(iii) learn how to construct meaningful and informative scientific studies using models;

(iv) learn how to communicate effectively about quantitative logic and results.

In this vision, the online library would have four types of resources for students (Fig. 3), contributed in the form of modules by a broad diversity of the scientific community. While we anticipate the flexibility to include variations, the general expectations for a contributed module to this library would include:

(i) Executable models: These are relevant to organismal function or interactions of organisms with higher and lower levels of biological organization. These models would typically be implemented in Jupyter notebooks that provide self-guiding background including modeling assumptions, usage instructions, and links to activities and useful references. Ideally, these models would be actual research tools used in (or at least based on) published literature, with clear and intuitive interfaces with minimal learning curves.

(ii) Activities: In which students are guided through examples of systematic and informative ways to use a specific model to generate and test hypotheses about organism function. Activities not only serve as demonstrations of a model's utility, but also present open-ended topics for independent inquiry and quantitative critical thinking.

(iii) Quick explainers: These are brief summaries of jargon terms, concepts, or methods referred to in the executable models or activities. The goal of quick explainers is to provide concise background, with information that is correct but abbreviated, with links and references to more comprehensive treatments of relevant subjects. Like “Tool Tips” in many applications, the intent is that quick explainers get students “unstuck” when they encounter unfamiliar topics in the pursuit of a modeling activity but are short enough in presentation so as to minimally disrupt that activity.

An additional element of this library would be a roadmap table, a searchable database that organizes library entries to facilitate finding models, activities and background relevant to a given organizational level, organism, conserved quantity, mechanism, or computational platform.

## Examples of web-based models for organismal biology teaching

We close with examples of Jupyter notebook-based models that illustrate some elements we envision for more quantitatively-oriented teaching, drawn from two very different subdisciplines within organismal biology. These examples have several traits in common:

(i) They present models actually used as research tools.

(ii) They have intuitive parameters (and not too many of them) as inputs.

(iii) They have visually clear graphical outputs as well as intuitive numerical outputs (and not too many of them).

(iv) They are implemented online and can be used on any browser (including a tablet) through binder.

(v) They describe mechanisms that link organism function to higher and lower levels of biological organization.

(vi) They present students with a new investigative tool to systematically investigate a long-standing biological “big question.”

Each of these traits is significant in making these notebooks suitable for engaging and empowering organismal biology students.

The first of these examples is a biomechanics investigation of the swimming performance of early stage marine invertebrate larvae. Several lines of evidence, including both habitat-level analyses of predation risk and developmental studies of cell lineages, suggest that swimming—specifically, the ability to maintain orientation and upwards movement—is a key trait in larval development. Swimming performance is strongly influenced by larval size and morphology, linking it to fundamental life history traits such as egg size and reproductive investments, feeding, predator avoidance, development time in the plankton, and transport by currents during development within and between habitats. Many larvae develop complex and fascinating morphologies that students find compelling. This model highlights to students that early stage embryos and larvae must perform many of the same tasks with only simplified morphologies, such as blastulae and gastrulae.

**Fig. 4:**
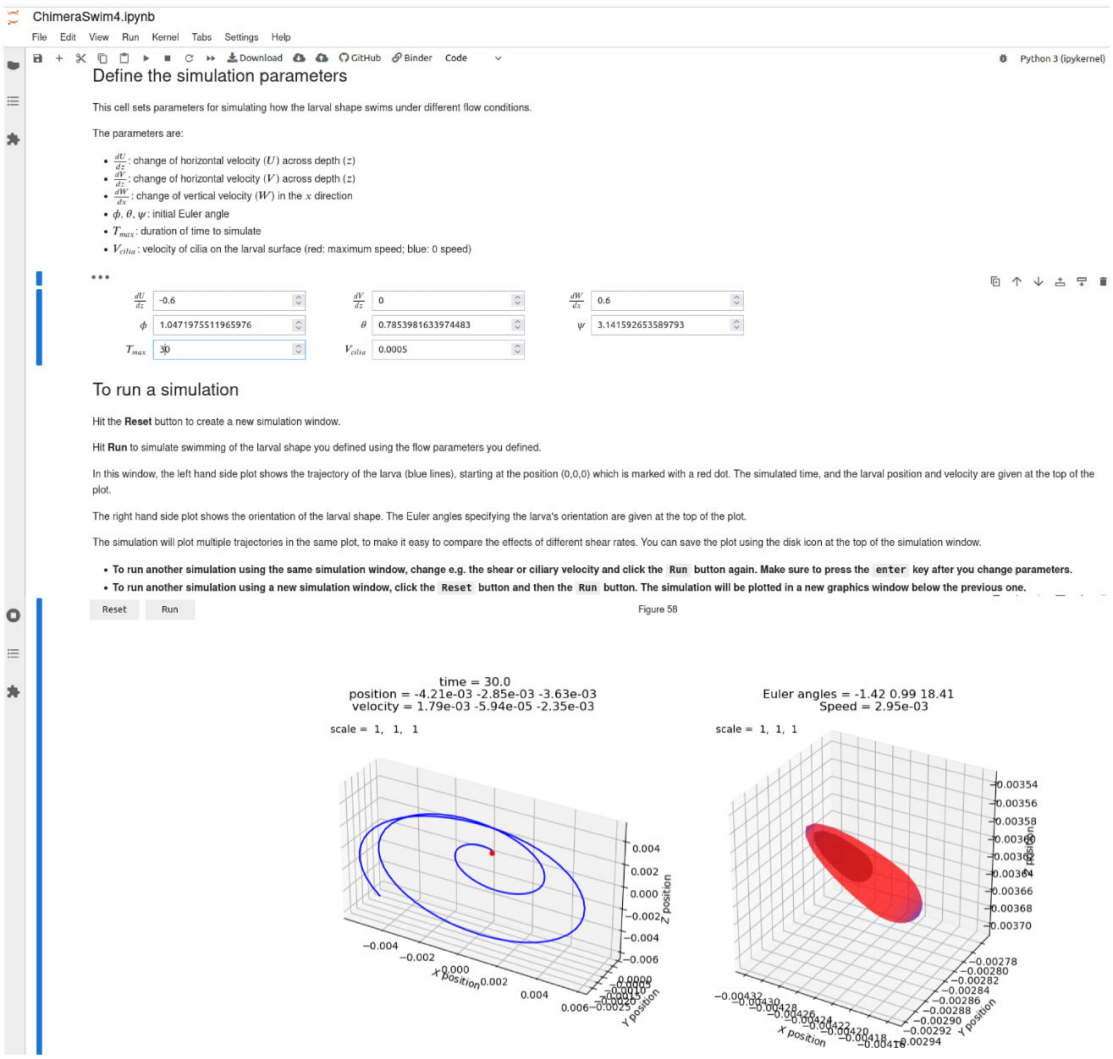
Examples of graphics and other content presented to students by the embryo swimming model, as implemented within Jupyter notebooks. In particular, this montage shows screenshots of what a student sees when using this notebook—self-contained background and usage instructions, simple interfaces for selecting parameters and visually engaging presentation of numerical results—all of which facilitate active learning and acquisition of modeling skills by organismal biology students. Some text may be difficult to read without high magnification, but even at low magnification the contrast of the Jupyter notebook framework with traditional command line inputs and outputs is clear. Elements of this user interface for simulating swimming in shear and rotating flow are shown, including the textboxes for specifying flow (in this case, pure rotation) and other simulation parameters (duration, ciliary velocity, initial orientation). At the bottom is the model output, including visualizations of larval trajectory, position, and orientation (that are played as a movie in real time), and numerical metrics of position, orientation, and velocity. In this example, the larva is able to maintain an inclined but stable orientation as it is advected in the rotating flow.

**Fig. 5:**
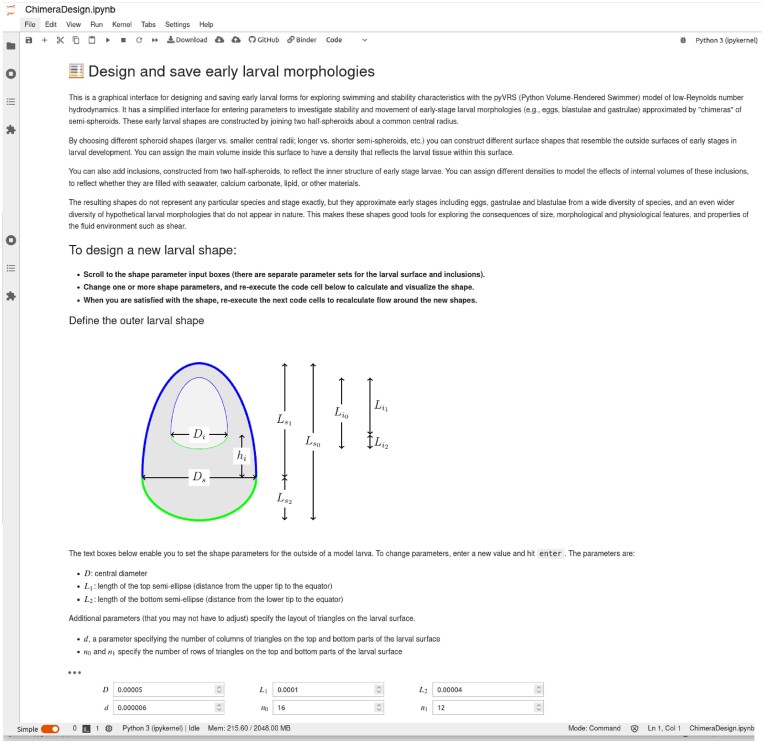
The Jupyter notebook interface for the embryo swimming model, showing tools provided to students enabling them to “design” early larval shapes by mimicking traits of extant species or inventing hypothetical ones. The notebook also presents a 3D rendering of the embryo morphology and, as an option for interested students, the numerical gridpoints used in the fluid dynamics calculations (not shown). This notebook enables students to investigate effects on swimming performance of specific changes in the size, morphology, and composition of early stage larvae. Students use this notebook in conjunction with the notebook in Fig. 4, to vary larval traits in the context of environmental variations (e.g., turbulent near-surface habitats vs. quiescent deep-water habitats). Working with this model helps students to understand scenarios under which swimming requirements impose benefits or constraints on larval size and shape, and to speculate about implications for life history evolution, species ranges, and other large time/space scale processes.

Most early stage larvae are sufficiently small and slow that their swimming is characterized by low Reynolds numbers (*R*), enabling a fluid dynamics model that is computationally tractable enough to be used in experiential learning by students. The model assumes, additionally, that larvae are automatons (lacking behavioral responses to orientation), that cilia act to generate a tangential velocity characterized by a “ciliary velocity” parameter, and for simplicity that morphologies can be constructed from “chimeras” of joined semi-spheroids. A key attribute of this model is that its implementation in a Jupyter notebook makes it self-explanatory, intuitive to operate and visually engaging (Figs. 4 and 5). For example, the morphology of a blastula or gastrula can be mimicked with one chimera of this type representing its outer surface and another chimera representing its blastocoel.

Students begin modeling activities by considering motivating questions such as:

(i) Which features of larvae and eggs reflect mechanical constraints on swimming performance?

(ii) Which features reflect other biological requirements? For example: Habitat selection (speed, orientation), feeding, encounter rate with predators (or sperm if an egg), energy usage, energy storage (amount and material), respiration (diffusive transport), or hydrodynamic signaling.

(iii) What tradeoffs are there in larval design that may reflect conflicting requirements?

Students are initially asked to consider metrics of swimming success most relevant to a larva, and to use their chosen metrics to assess the swimming performance of a default larval morphology: How does it perform across systematic variation in intensity of vorticity or shear? Students are then provided with estimates of turbulent intensities in different environments (deep ocean, near-surface, etc.) expressed in terms of characteristic vorticity and shear, and are asked to discuss what their observations suggest about environments in which the default shape can or cannot swim successfully. Students can then consider the impacts of size, by isometrically increasing and decreasing larval size, and examining the performance of these larger and smaller individuals in rotating and shear flows in the context of other effects of size (e.g., a size—number tradeoff in maternal allocation). Finally, students are invited to design new larval shapes, based on existing larvae from specific habitats or hypothetical larvae that are not known to exist, considering how these larvae might reflect specialization (or lack of it) for challenging environmental conditions.

### Exploring cholera disease dynamics and mitigation strategies

**Fig. 6:**
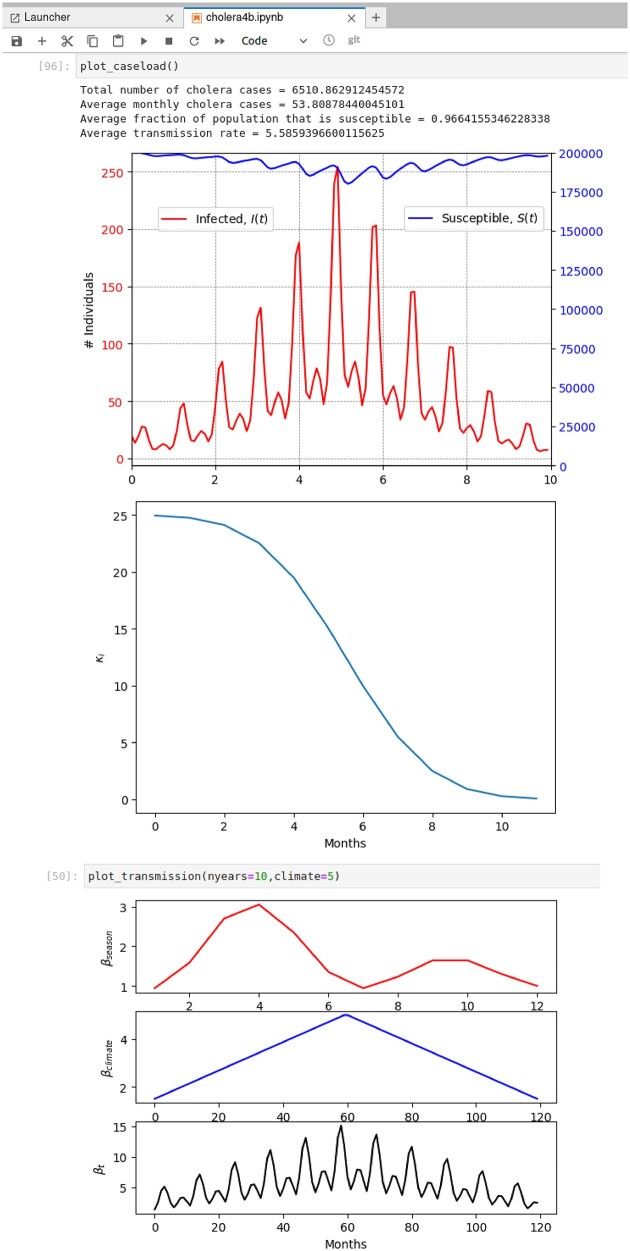
Screenshots of graphics and numerical output presented to students by the cholera model from Koelle et al. (2005), as implemented within a Jupyter notebook. The top plot shows key results from a cholera simulation scenario: Time series of infected and susceptible individuals (note different axes) across rapid seasonal variations and decade-scale “climatic” changes roughly corresponding to El Niño Southern Oscillation fluctuations. After studying and understanding these time series with default parameters, students consider the underlying environmental and physiological mechanisms. The middle plot visualizes how immunity (K*_i_*) decays over time in individuals who have recovered from a cholera infection, an intuitive graphical presentation of a concept from epidemiology (“immune period”) that students often find confusing and mathematically complex. The lower plots show how fluctuations due to precipitation variations on seasonal and climatic timescales impact total transmission rate (β_t_), which corresponds to the environmental contribution to the basic reproductive number, ${R_0}$. Students complete the exercise by manipulating these driving mechanisms, considering the potential effects of alternative mitigation strategies, such as increasing the immune period by improving general health or reducing transmission by improving sanitation.

Our other example is an epidemiological investigation of cholera dynamics in humans, based on estimates of periods of immunity after infection, asymptomatic ratios, strain dependence, and other factors, from Koelle et al. (2005) using data from Matlab, Bangladesh (Fig. 5). These estimates were derived using parameter fitting for an epidemiological model. The Jupyter notebook we used for teaching recreates this model in a form that facilitates exploration by students of disease dynamics with alternative parameters. Again, the effectiveness of the modeling exercise stems from graphical output (Fig. 6) that immediately and intuitively presents the implications of otherwise opaque mathematical functions. In the activities, students are first asked to consider the possibility that parameter estimates by Koelle et al. (2005) are slightly (or even wildly) erroneous. This motivates an initial sensitivity analysis, assessing how much the magnitude and timing of cholera outbreaks are altered by the immune period, and by fluctuations of transmission (${R_0}$) across seasons and multiyear “climatic” fluctuations such as El Niño and La Niña.

Students are then asked to report to a hypothetical NGO about the relative merits of three potential interventions: Improving sanitation to reduce transmission surges; vaccination to reduce the pool of susceptible people; or, improving nutrition and basic health to extend the immune period. For each intervention, students are asked to construct quantitative estimates of reductions in cholera caseloads, given assumptions about the impacts of mitigation on the relevant epidemiological parameters.

## Conclusions

Our goal here has been to highlight a fortunate synergy between science agendas at three levels: Core Ideas, Scientific and Engineering Practices and Crosscutting Concepts defined for K-12 education by the NGSS; core competencies for biologists identified by the AAAS's Vision and Change document; and grand challenges in organismal biology as articulated by the SICB and others. Each of these agendas embraces an emphasis on quantitative critical thinking, particularly on understanding and correctly using mathematical models as tools for generating and testing hypotheses, for making inferences and predictions, and for communicating between diverse communities that frame questions differently and have distinct vocabularies. As scientific knowledge has expanded at the lowest levels of biological organization (e.g., gene networks) and at the highest levels (e.g., ecosystems), this knowledge has increasingly been codified, communicated, and exploited using systems-type models. Organismal biology—the study of the organisms that carry these low-level systems and operate within the high-level systems—has, by comparison, invested less in systems modeling. We argue that there is much to be gained, in terms of advancing the science of organisms and of making our knowledge more broadly useful in other fields, by deepening this investment.

We present some suggestions about implementation of model-informed teaching approaches for the next generations of organismal biology students. Our suggestions stem in part from the expectation that, as students arrive with long-term exposure to curricula influenced by NGSS and Vision and Change, they will be better prepared to conceive and communicate their own research and teaching in modeling terms. We highlight the suitability of recently developed open source, online platforms for web-based scientific computing such as Python, Jupyter notebooks, and executable books. These platforms make it possible, for the first time, to envision a community-driven library of systems-type models of organisms, presented in context on browser pages with information about the model, usage instructions, additional references, and guided activities for learning. In particular, these platforms make it possible for individual investigators to post their own research models in a no-cost, highly accessible format that can be used by anyone with a browser and an internet connection. Such models, and the context in which they are embedded, enable users to interact with and critically assess models constructed and presented by experts. By removing the prerequisite for advanced coding or mathematical skills, a model library of this sort could mitigate one of the most limiting aspects of modeling for many organismal biologists—the long technical training and time required to devise these models from scratch.

## Data Availability

No data were collected for this paper

## Notes

From the symposium “Modeling organismal responses to changing environments” presented at the annual meeting of the Society for Integrative and Comparative Biology, January 2–6, 2024.
